# New Community and Sociohealth Challenges Arising from the Early Diagnosis of Mild Cognitive Impairment (MCI)

**DOI:** 10.3390/jpm13091410

**Published:** 2023-09-20

**Authors:** Carolina López, Miren Altuna

**Affiliations:** 1 Fundación CITA-Alzheimer Fundazioa, 20009 Gipuzkoa, Spain; 2 Osakidetza, Organización Sanitaria Integrada (OSI), 20690 Gipuzkoa, Spain

**Keywords:** mild cognitive impairment, Alzheimer’s disease, stigma, caregivers, personalized medicine, advance care

## Abstract

Population aging increases the risk of developing neurodegenerative diseases that cause cognitive impairment. Advances in clinical practice and greater social awareness of the importance of cognitive impairment have led to an increase in the number of people with early diagnosis, predementia. Increasing access to biomarkers to assess whether Alzheimer’s disease (AD) is the underlying cause of mild cognitive impairment (MCI) has undoubted clinical benefits (access to potentially disease-modifying treatments, among others) but is also responsible for new social–health care challenges. Understanding the psychosocial impact of a diagnosis of MCI due to AD or another neurodegenerative disease is essential to create future strategies to reduce the emotional overload of patients, their risk of discrimination and stigmatization, and to favor their social inclusion. We present a narrative review of the diagnostic process of mild cognitive impairment in clinical practice, with a holistic person-centered approach, and discuss the implications of such diagnosis (benefits and risks) and strategies on how to address them.

## 1. Introduction

Cognitive complaints are an emerging reason for consultation in both primary care and neurology [1]. This is explained both by an aging population and a greater social awareness of the importance of early diagnosis of cognitive problems. In recent years, there has been an increase in the number of clinical cases diagnosed in predementia stages of cognitive impairment in developed countries, as well as in the number of patients with early etiological diagnosis supported by the use of biomarkers that define the biology of the underlying cause [2,3]. Recently, there have also been promising advances in treatment not only symptomatic but also potentially modifying the clinical–biological course of Alzheimer’s disease (AD) [4,5,6], one of the most frequent and severe causes of cognitive impairment. However, improving diagnostic capacity and making such diagnoses early has also brought new social and healthcare challenges (stigmatization risk, need to empower patient decision-making, etc.), especially in those patients with a diagnosis of progressive and irreversible deterioration.

We conducted a narrative review on the importance not only in terms of health but also in decision-making and in ethical, social, and emotional implications derived from an early diagnosis of cognitive impairment in the predementia stage, focusing on the benefits and new challenges derived from it. Detecting the needs of these patients is key to empowering the patient and creating a personalized and integrated social–health care plan. This information is crucial to propose future accurate psychoeducational and socioemotional interventions to mitigate the potential negative effects of an early diagnosis of mild cognitive impairment (MCI) and to enhance the well-known benefits.

## 2. Methodology

**Figure 1:**
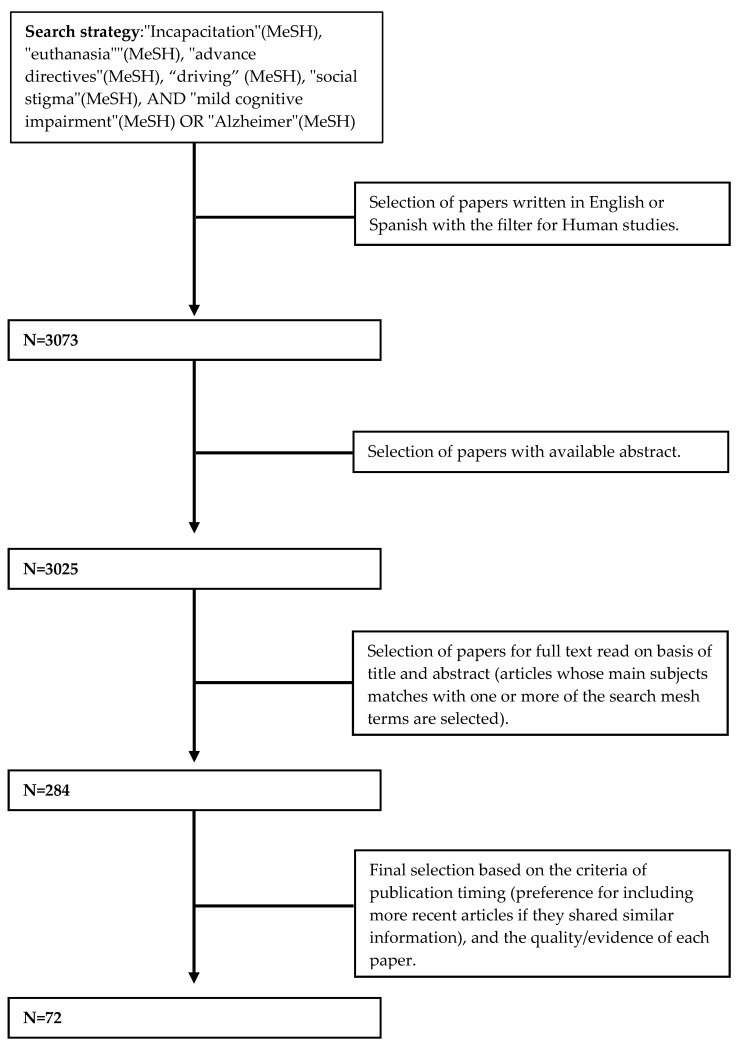
Flowchart of the research strategy.

We performed a literature review using PubMed and Web of Science (WOS), with a search strategy with the following MeSH terms (Medical Subject Headings): “Incapacitation”, “euthanasia”, “advance directives”, “driving”, “social stigma”, “mild cognitive impairment”, and “Alzheimer” (Figure 1). We did not apply any time restriction, and we included original articles and review articles with data from human subjects (exclusion of papers in animals only) written in English or Spanish available on 10 July 2023. We selected articles with abstracts available in PubMed or WOS. After reading the titles and abstracts, those papers that met the eligibility criteria were selected for full-text revision. Those papers specifically dealing with MCI or prodromal AD from a clinical or psychosocial perspective were included in this review. To ensure an extensive search, we also manually searched the reference lists of all included articles. The final selection of articles included was based on the criteria of publication timing (preference for including more recent articles if they shared similar information) and the quality/evidence of each paper (preference for original articles and meta-analyses over narrative reviews and case series). Therefore, older and/or less scientifically rigorous articles that did not provide additional information were not included.

The review is divided into four main sections: (1) mild cognitive impairment (MCI) concept explanation, (2) benefits of an early diagnosis of MCI, (3) socio-health challenges due to early diagnosis of MCI, and (4) we finalize with a section about the strategies for reducing social stigma and behavioral–affective impact derived from an early diagnosis of MCI.

## 3. Mild Cognitive Impairment (MCI)

Cognitive abilities often decline with age. Fortunately, we are able to properly identify these changes that are not related to pathological aging and not misdiagnose the existence of a mild cognitive impairment (MCI) [7].

The presence of cognitive decline affecting at least one cognitive domain on neurocognitive testing (scores lower than 1.5 standard deviations compared with normative cut-off values of validated neuropsychological tests in comparable populations according to age and educational level) but without significant impact in instrumental activities of daily living is classified as MCI [8,9]. Therefore, to make a diagnosis, it is necessary to explore the complaints of the patient and informant, to perform neuropsychological tests, both screening and some specific ones, and to pass questionnaires of functionality. However, self-awareness of impaired cognitive performance is not necessary to define MCI [10]. The concept of MCI is very important to differentiate it from that of self-estimated or subjective decline in cognitive capacity, with expected or unimpaired performance on neuropsychological tests [11]. The risk of progression of cognitive impairment to dementia (repercussion on previous personal autonomy) in the context of MCI is estimated to vary between 5 and 15% per year, depending, of course, on the etiology [10].

### 3.1. Prevalence of MCI

The prevalence of MCI varies significantly depending on the definition and diagnostic criteria used (neuropsychological evaluations with more or less demanding cut-off points) and on whether they are population-based and/or limited to a specific healthcare setting. However, estimates suggest that more than 6% of the population over 60 years of age would have MCI [12] and that this could be as high as 20% in those over 80 years of age [13].

### 3.2. Diagnosis of MCI

The diagnosis of MCI is clinical and requires an assessment of the cognitive status of each individual adjusted to their age and academic–professional skills. The brief cognitive screening tests available in clinical practice are mostly designed for a diagnosis of dementia rather than MCI and for a population with an academic level usually lower than the current one [12]. Furthermore, the neuropsychological batteries currently used, although they are more sensitive for detecting MCI than brief cognitive screening tests, may have cut-off points validated in a population that is not well phenotyped clinicobiologically, have a lower diagnosis performance for nonamnesic predominance MCI, and/or is not representative of the population currently being tested [9,12]. It is suggested, therefore, that there is an indication to create new normative values adapted to the population at greatest risk of developing cognitive impairment at the current time (both for brief cognitive testing and for comprehensive neuropsychological batteries) and that the classification between healthy controls and persons with cognitive impairment (MCI or dementia) should be accompanied by an exhaustive biological categorization.

### 3.3. Etiology of MCI

**Figure 2:**
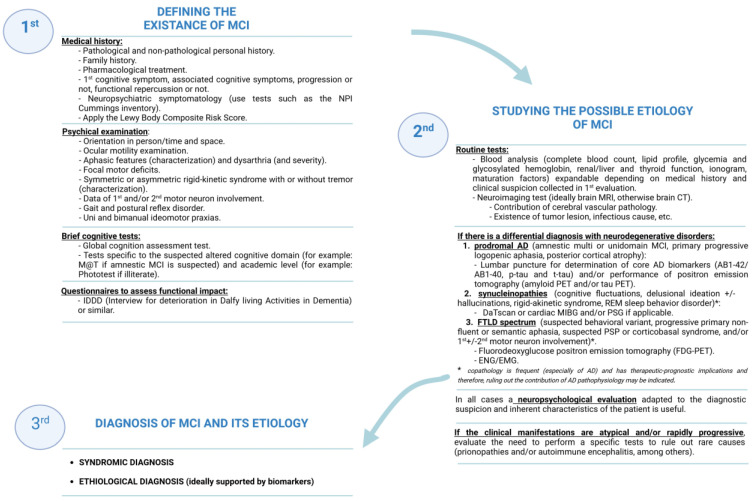
Summary of the diagnosis process of a patient with cognitive complaints: MCI, mild cognitive impairment; M@T, memory alteration test; MRI, magnetic resonance imaging; CT, computerized tomography; FTLD, frontotemporal lobar degeneration; PSP, progressive supranuclear palsy; ENG, electroneurogram; EMG, electromyogram. Created with biorender.com.

MCI can be either stable or progressive and of both neurodegenerative (Alzheimer’s disease, Lewy body disease, frontotemporal lobar degeneration, Parkinson’s disease, prion disorders, etc.) and non-neurodegenerative causes (affective disorders, vascular etiology, drug-induced, etc.). In turn, the outcome of MCI varies considerably depending on the underlying cause [1]. The profile of cognitive impairment with involvement of one or more cognitive domains, with amnesic predominance or not, and the coexistence of neuropsychiatric or motor symptoms may help to suggest the etiological diagnosis of MCI, but in no case does it replace the application of neuroimaging and/or biochemical biomarkers currently accessible for use in clinical practice (Figure 2).

The first step is to confirm whether there is a modifiable and treatable cause of MCI. Secondly, it is to assess whether or not there is a progressive disease and what is the estimated prognosis of such pathology. Early etiological study is vital in order to be able to have such information and initiate a potential pharmacological or nonpharmacological treatment. The etiological diagnosis of subjective cognitive complaints must evaluate the coexistence of neuropsychiatric symptoms, destabilizing medical comorbidities, and the use of psychotropic drugs, among others, but in no case biomarkers of the physiopathogenesis of AD and/or synucleinopathies. Diagnosis in preclinical or presymptomatic stages is not supported in the context of clinical practice as long as there are no disease-modifying treatments approved for presymptomatic stages of the disease [1].

Alzheimer’s disease (AD) is the most common cause of MCI of neurodegenerative etiology, but prodromal AD diagnosis requires biological confirmation of the existence of amyloid and tau pathology (CSF AT(N) biomarkers or PET amyloid) [8,14]. In addition, research criteria potentially applicable in clinical practice for the diagnosis of prodromal Lewy body disease [15], a common cause of MCI along with idiopathic Parkinson’s disease, have recently been published. The copathology of both AD-Lewy body disease and AD-vascular pathology is very frequent, and the coexistence of both pathologies seems to have a poorer cognitive and functional prognosis [16]. Therefore, defining not only the primary etiological diagnosis but also the existence or not of copathology, is of great importance. The coexistence of affective symptoms that may have an independent negative impact on cognitive performance should also be explored, as well as the chronic use of psychotropic drugs and the quality of sleep since all of them may be modifying factors. The potential coexistence of nonmotor focal epileptiform seizures that may be confused with cognitive–conductual fluctuations should also not be forgotten, and their nontreatment may result in a more torpid clinical course [17].

## 4. Benefits of an Early Syndromic and Etiological Diagnosis of MCI

Ensuring universal early access to the syndromic and etiologic diagnosis of MCI with the highest possible degree of certainty during life (definitive diagnosis requires *postmortem* assessment) has undoubted benefits for the patient, their relatives, and their present and future caregivers: (1) empowerment of patients with intact intellectual capabilities for decision-making about their present and future care and life choices (the possibility of adjusting professional activity, deciding on their future place of residence, etc.) [18,19]; (2) to ensure access to pharmacological and nonpharmacological treatments (including psychoeducational, cognitive training, and dietary and physical activity interventions) with a potential disease-modifying effect on the clinical course of the disease; and (3) facilitating access to clinical trials and/or other research studies according to the patient’s own wishes [20,21]. Specifically, one of the potential benefits, extremely relevant in the case of MCI due to neurodegenerative disorders such as AD, is the opportunity to write the advance directives document. This is an appropriate way to determine the patient’s wishes about testing and treatment before they are incapable of expressing them at the severe stage of dementia [22].

### 4.1. Pharmacological and Nonpharmacological Treatment of MCI

First of all, an early diagnosis of MCI allows the option of offering the patient an individualized cognitive training/stimulation plan. Ideally, this should be adapted not only to the patient’s current cognitive status but also to their academic–professional life context and should be carried out in their native language. A holistic approach with the promotion of brain-healthy lifestyle habits, not only cognitive stimulation, but also its association with physical activity [23], good adherence to a healthy diet, such as the Mediterranean diet [24,25,26], and the preservation of socioemotional health [27], has been shown to help reduce the risk or at least the speed of progression of cognitive impairment in different causes of MCI [28]. However, the benefit goes beyond promoting lifestyle changes since it allows, among other things, the optimization of pharmacological treatment of frequent medical comorbidities, for example, the adjustment of anxiolytic and antidepressant treatments for those with a better cognitive and motor safety profile.

There are also specific treatments with symptomatic and potentially clinicobiological course-modifying benefits for some specific causes of MCI. Specifically, symptomatic treatments such as acetylcholinesterase inhibitors have been available for over 20 years, approved by both the Food and Drug Administration (FDA) and the European Medicine Agency (EMA), for use in cognitive impairment due to AD, mixed vascular AD, and in the case of synucleinopathies. Although these treatments with potential symptomatic benefits on mood and behavioral disorders have been postulated for use in MCI due to AD and MCI in the context of synucleinopathies, they have only been proven effective in the case of dementia. Therefore, although their use is common in clinical practice in predementia stages, this is not supported by clinical practice guideline recommendations [29,30,31].

In recent years, the FDA has granted approval for use in prodromal AD and initial AD dementia to Lecanemab [5] and conditional approval to Aducanumab [4,32] for use in the same clinical context. In addition to being antiamyloid immunotherapy, both share biological efficacy in removing brain amyloid. Lecanemab has also demonstrated a significant but modest clinical benefit in global cognition, even in the prodromal stages of AD. In 2023, Donanemab [6], another antiamyloid drug with a similar mechanism of action, also presented positive clinical–biological results similar to those of Lecanemab. Currently, the decision of regulatory agencies on its approval for use in clinical practice is pending. These new biologic treatments represent an opportunity for hope for all patients with prodromal AD, but also an undoubted sociohealth challenge since they require a radical change in the system of care for cognitive complaints and the etiological study of these complaints (biological confirmation of AD pathology is mandatory). As these are also medications with possible adverse effects that require clinical and neuroimaging monitoring throughout treatment, and because they are administered intravenously, it is essential to inform each potential candidate of the individualized benefit–risk ratio. It is essential that the patient makes the decision to treat or not to treat freely and that the clinician does not assume a paternalistic attitude at the time of prescription.

### 4.2. Advance Directives

The advance healthcare directives or advance directives document is intended to be everyone’s living will. In this document, each person freely and with full intellectual capacity decides on their present and future health and social–health care and designates the person who will be their representative in order to respect their wishes in the case they are unable to do so [33]. In recent years, progress has been made in creating public electronic registries of advance directives accessible to all healthcare and social–health care personnel, with access controlled to always ensure confidentiality with maximum possible rigor.

Advance directives are designed to solve the dilemma of decision-making in the case of patients who are no longer capable of exercising self-determination and to avoid paternalistic decision-making in such individual cases [19,34,35]. It is, therefore, a crucial element for personalized medicine and to dignify the social and healthcare of the most vulnerable people.

During the process of communicating a diagnosis of MCI, especially if it is of a progressive neurodegenerative nature, the clinician must raise to the patient the possibility of writing or modifying the advance directives. Advice must be given on its drafting, specifying the wishes/decisions that may be defined in the document and the specifications that a potential representative must have, but in no case must the clinician make decisions about the patient’s future, respecting their will according to their clinical and vital context [33,36]. Although such recommendations should be routinely made in clinical practice, it is believed that this is not universally implemented, and there are no registries that allow us to know whether the diagnosis of MCI and, specifically, prodromal AD supported by biomarkers, implies a greater probability of drafting an advance directives document or not at the present time. The universal implementation at the population level of advance directive documents requires concrete policies and actions to make advance directives known and effective [35].

In recent years, in various European countries, including Spain, the advance directives document not only makes it possible to define the patient’s refusal of access to certain healthcare services (mechanical ventilation, enteral nutrition, among others) in the case of advanced cognitive and functional deterioration, but also to establish whether the patient would wish to have access to assisted suicide. Among the causes of assisted suicide with advance request, neurodegenerative diseases stand out. It is, therefore, crucial that clinicians inform patients about the possibility and its implications when they are capable of making their own free decisions [37].

### 4.3. Protection of the Dignity and Integrity of the Person: Legal Perspective

Besides the advance directives document, there are other legal mechanisms to ensure the dignity of the patient and respect for their wishes, such as proxy directives, including power of attorney and healthcare surrogate, and the person designed as the guardianship substitute:

- A power of attorney is useful when a person is unable to perform complex specific tasks without assistance (financial decisions, for instance) and they are not legally incapacitated [38];
- Instead, a healthcare surrogate is a person authorized via the Designation of Health Care Surrogate form to make medical decisions when the patient is not able to do so;
- A guardianship substitutes decision-makers, depending on the type of guardianship imposed by the court, and has the power to perform financial, personal, legal, and healthcare choices for their ward. Guardians of property are responsible for the financial assets of the ward, whereas plenary guardians are responsible for every aspect of the ward.

All of these mechanisms are part of advance care planning as well as advance directive documents and should be properly explained by the clinicians to the patients and their relatives in the early stages of the disease [19,38,39]. Each patient can express the person whom they would prefer to be their future guardian in their advance directive documents.

Formal recognition of the rights of people with cognitive decline, both with MCI and dementia, independently of the cause and premorbid individual socioeconomic status, through legislation and regulatory processes will help reduce discriminatory practices and, thus, ensure care and protection measures in the advanced stage of the disease, in which the capacity for judgment and self-determination are impaired, precluding control over their own decisions [33].

## 5. Sociohealth Challenges due to the Early Diagnosis of MCI

Early diagnosis of MCI of neurodegenerative etiology, including prodromal AD supported by biomarkers, is a reality of great relevance. With the development and application in clinical practice of more accessible and accurate diagnostic biomarkers (plasma biomarkers) [40], it is expected that the number of cases of AD diagnosed in prodromal or MCI stages will increase exponentially in the coming years. Unfortunately, the early diagnosis of progressive neurodegenerative disorder raises a number of ethical concerns, such as social stigma, psychosocial damage, driving fitness, available social care services, and lack of adaptation to the new scenario [41]. Therefore, involvement and counseling of the patients and their relatives before and after a syndromic and etiological diagnosis has been established as essential [1].

This new reality, no longer a remote futurist one, requires: (1) ensuring universal and equal access to tests that allow early and accurate diagnosis of MCI and its etiology, regardless of the individual’s socioeconomic status and geographic location; (2) training clinicians involved and creating guidelines/protocols to optimize the process of communicating diagnostic results in a personalized way according to the vital reality of each patient; and (3) adapting laws, regulations, and professional practices to the diagnosis of prodromal AD [42] and, maybe in the near future, prodromal Lewy body diseases (currently only research criteria and not clinical criteria available).

Improvement in the training of primary care and neurology clinicians, most accessible noninvasive and less expensive biomarkers, improvement in brief cognitive test cut-offs (more demanding for increasing sensitivity), and modernization of neuropsychological assessment, as well as the creation of specific psychoeducative programs, will allow for a reduction in the stigma and discrimination of people with prodromal AD [1,21]. On the other hand, advances in the potentially modifying treatments of AD, applied in early predementia stages, will help to reduce the mistaken belief that early diagnosis carries a risk of unnecessary overmedicalization and increased risk of emotional overload for both the patient and their relatives.

However, despite progress in improving clinical practice, several undesirable consequences can occur after the disclosure of an early diagnosis. These implications are related to work, driver’s license, insurance, and stigmatization [18]. Therefore, it is essential to address these problems by implementing a support and guidance plan from the moment of the diagnostic disclosure.

### 5.1. Stigma and Risk of Sociolabor Exclusion

Stigma is defined as negative stereotyped beliefs, feelings, and behaviors [43]. It is a complex concept and may occur at the individual, interpersonal, family, societal, and institutional level. Stigma, when fully enacted, excludes patients from participation in everyday life or “full personhood” [44]. In other words, it leads to the depersonalization of the ill person, reducing them only to the diagnosis. Relating a patient with a specific “disease label” favors simplified, exaggerated, and inaccurate interpretation of the symptoms and signs of a patient [45,46]. In addition, stigma also contributes to avoiding help-seeking behaviors, delaying the diagnosis, and reducing access to proper health and social services [47].

AD and other neurodegenerative disorders causing cognitive decline are stigmatized health comorbidities that cause significant negative effects on patients as well as their caregivers, such as low self-esteem, isolation, poor mental health, and decreased quality of life [47]. Stigmatization of the family and/or caregiver of a cognitively impaired patient is a clear cause of stigma by association and is not without risk. It favors social and occupational discrimination of family and caregivers [44,48].

Patients recently diagnosed with MCI, mainly neurodegenerative MCI, including prodromal AD, are at risk of being excluded from the labor market, although they could continue to work with minor adaptations of their activity or increased supervision in the more complex tasks. The risk of exclusion also goes beyond the labor market since the diagnosis of a neurodegenerative disease, even at a very early stage, currently also hinders access to certain sociohealth services, such as private health insurance, and favors discrimination in healthcare, with a more restrictive therapeutic approach to other associated medical conditions [49,50,51]. Therefore, research into MCI and specifically prodromal-AD-related stigma and developing support programs is an important research area that is currently underinvested.

### 5.2. Impact on Socioemotional Health

Cognitive impairment, and specifically AD from its earlier symptomatic stages, seems to be an independent risk factor for the development of neuropsychiatric symptomatology. Not only the detriment of cognitive performance with respect to premorbid conditions and the possible diagnosis of a progressive, irreversible neurodegenerative disease such as AD is responsible for an increased risk of developing affective symptoms such as apathy, anhedonia, and/or anxiety [52]. The existence of affective symptoms may also negatively influence sleep quality and quantity, and this, as well, can worsen the emotional status of a patient and their own cognitive performance. Surprisingly, it seems that anxiety may decrease once the etiological diagnosis of cognitive impairment is performed. Uncertainty and fear of the unknown could, therefore, facilitate independently a higher burden of affective symptoms [18,21].

In addition, poorer life satisfaction and quality of life have also been reported in an observational cross-sectional study in 21 memory clinics in Spain [53]. Indeed, stigmatization, a greater risk of sociolabor exclusion, and uncertainty about the future increase the probability of presenting moderate–severe affective symptomatology [18,47,54]. Furthermore, the gradual loss of cognitive or intellectual abilities is perceived as a massive threat to personal integrity, autonomy, and identity, more intensively in the early stages of neurodegenerative disorders such as AD [55]. Fortunately, we can modify the latter by means of nonpharmacological psychoeducational measures applied to the patient (emotional support and guidance), their relatives and/or caregivers, health professionals, and the whole of society [47]. Sometimes, the use of psychotropic drugs is required, but they should be used only when nonpharmacological measures are not effective and always at the lowest dose possible and during the smallest amount of time. Drugs to be used should be chosen carefully according to the patient’s comorbidities and potential negative impact on their cognition or motor abilities.

### 5.3. Repercussion on Complex Regulated Activities: Driving

Individuals with an AD diagnosis and risk of dementia are considered vulnerable to discrimination, affecting their professional position, insurance fees, legal status, civil rights (driving and voting), and financial capacity.

The presence of cognitive impairment may influence driving capacity because safely driving requires the integration of multiple cognitive domains (visual perception, executive function, episodic, semantic, and procedural memory, as well as complex attention and processing speed) and sensory and motor functions. There are no well-defined criteria to establish as objectively as possible when a person with cognitive impairment should stop driving [56]. Withdrawing this right prematurely can lead to discrimination and stigmatization, and doing so too late can pose a risk to their own safety and that of everyone else. It would, therefore, be helpful and necessary to reach a consensus among professionals to properly assess the ability to continue driving.

Fortunately, some drivers, mainly females, with MCI are able to recognize their cognitive limitations and adjust their driving accordingly [57]. Indeed, a significant proportion of people with MCI stop driving within 3 years after diagnosis. Characteristics such as age, cognition, functionality, and changes in these over the next 6 months predict driving cessation [58], and depressive symptoms may mediate driving ability to a greater extent than in the general population [59,60]. Alternatives to hard decisions, such as withdrawing a driving license, could be to only allow driving during daylight hours or for short distances or below a speed.

There are several studies that aim to establish an adequate protocol to assess driving skills, and in these studies, neuropsychological tests are considered adequate, focusing mainly on executive functions [61,62] and the screening of visual and motor disorders [63]. The risk of accidents in both elderly with and without cognitive decline seems to be related to measurable conditions such as reaction time, speed variability, and lateral position variation [64]. Recently, it has been demonstrated that cognitive decline and driving education and accompanying decision aid demonstrate an efficacious solution for a diverse range of health practitioners to enhance their knowledge, confidence, and competence in supporting people living with dementia with driving retirement decisions [56].

In addition, an earlier diagnosis of cognitive decline, including MCI due to AD, may affect access to health insurance. Indeed, a diagnosis of a neurodegenerative disorder may make it difficult to access a specific health service (such as health insurance) or at least make them more expensive [21,65]. In turn, no proper legal directives have been created to favor the continuity of patients with MCI in the labor market. These aspects facilitate further discrimination and stigmatization of patients with MCI.

## 6. Strategies to Reduce Social Stigma and Behavioral–Affective Impact and Favor the Inclusion of People with MCI

**Figure 3:**
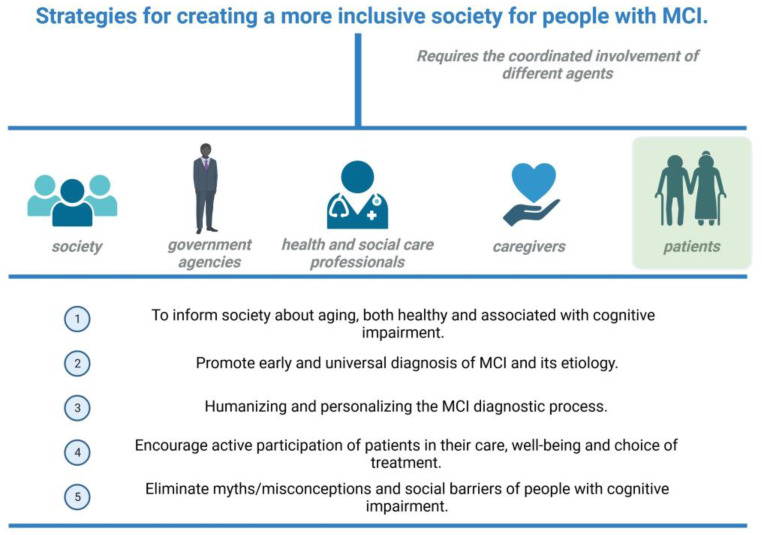
Summary of the strategies to reduce stigma and socioemotional burden to patients and caregivers. Created with biorender.com.

The process of diagnosis of MCI of neurodegenerative cause, and specifically of prodromal AD, must be guided by adequately qualified professionals not only at the health level but also at the psychosocial level. Healthcare professionals, at the moment of communicating the diagnostic results, should offer resources to access self-care and become or stay engaged with one’s community. That may be useful in order to prevent isolation and to improve the management of potential affective symptoms [47] (Figure 3).

### 6.1. Training for Healthcare and Social–Health Care Professionals and Caregivers and Social Awareness

Society’s lack of knowledge about cognitive impairment in the early stages, including prodromal AD, favors the risk of stigmatization and social discrimination of the patient. As a result, early access to social and healthcare resources is reduced, and there is a risk of infringing on the inherent rights of people with cognitive impairment [47]. It is surprising how, at the population level, the existence of cognitive impairment is still attributed to nonrational or nonscientific reasons. According to the World Alzheimer Report 2019, more than 20% of respondents believed that dementia is caused by external forces such as “bad luck” (21.7%), “providence” (8.7%), and “witchcraft” (1.9%) [50]. In turn, a recent study conducted in China reported that 44.6% of the participants, in a study on knowledge about AD, wrongly believed that cognitive decline and even dementia, including AD dementia, is part of the normal aging process [66]. To believe that cognitive decline, including AD, is a normal age-related process may further increase the perceived causal responsibility of cognitive and neuropsychiatric symptoms for the patient, increasing their emotional burden [67].

The level of knowledge about both cognitive impairment and AD, specifically both at the level of society and of the sociohealth professionals themselves, is clearly influenced by their level of education, access to different media, and place of residence (urban vs. rural) [66]. Therefore, demographic factors should be considered in early dementia interventions targeted at different groups.

There is robust evidence about the utility of population campaigns to reduce the stigmatization of patients with AD-related disorders. These campaigns must be designed specifically for the target population, using different communication channels and comprehensive language to achieve their objectives [47].

### 6.2. Patient Empowerment Strategies

An early diagnosis of MCI of neurodegenerative cause with an active role of the patient throughout the diagnostic process is essential. The clinical suspicion, indication of each test to be performed, and implications of the test result prior to its performance should be explained. Once the syndromic and etiological diagnosis of MCI has been established, it should be communicated to the patient, who should decide whether to communicate it to their relatives or others [1]. In addition to issuing the diagnosis and providing access to pharmacological and nonpharmacological treatment, the patient should be provided with understandable information (better if it is written material) about their pathology in their native language. Education about their present and future health condition is the best way to empower the patients [68].

Postdiagnostic support after MCI diagnosis must have holistic, integrated, personalized care of their patient and their relatives [69]. This support must be multidisciplinary and should be coordinated by neurologists or geriatricians if possible, and if not, by primary care physicians. Ideally, this person-center care should include [70] (1) individualized, goal-oriented care planning based on the preferences and the goals of the patient with cognitive impairment, (2) ongoing reassessment of care plan and preferences, (3) interprofessional team-based care with active continuous information sharing and accurate coordination strategy, (4) education and training provided to healthcare providers and for the patient and relatives, and (5) measurable outcomes with feedback from the person living with cognitive decline.

In order to provide good clinical assistance to patients with MCI, it is necessary to (1) timely identify and manage current and future needs and (2) design an integrated support plan [71]. For this purpose, it is essential to (1) inform the patient about the expected progression of the disease and available treatment options, (2) offer psychological, personalized support, (3) perform regular medical clinical reviews, and (4) facilitate general recommendations about adaptation at home, advise on diet, financial benefits, available social services, and so on.

### 6.3. Promoting Inclusive Policies

The creation of protocols for healthcare professionals to promote a reduction in the risk of stigmatization derived from a diagnosis of MCI is a necessity. Sociohealth policies that understand that the patient is not defined by the disease and that dignity and personalized care require a different sociohealth treatment than that granted in the usual clinical practice are crucial [47].

The promotion of public health strategies to improve the diagnosis of MCI, and specifically prodromal AD, should, therefore, not be limited exclusively to favoring universal and early access to a diagnosis based on biomarkers (already very relevant) but also to humanizing the care of people with MCI, which, in many cases, have a progressive and irreversible cause [72].

## 7. Conclusions

The diagnosis of MCI, including prodromal AD, is becoming more and more common in clinical practice, favoring the initiation of pharmacological and nonpharmacological interventions and, thus, increasing its benefits, as well as offering the opportunity for care planning. Despite these benefits, we cannot ignore the psychological, social, and legal impact of receiving a diagnosis. The next step in the process should be to implement comprehensive and holistic care based on individual needs, values, and wishes (person-centered care) (Table 1).

This recent clinical perspective introduces a new paradigm in which it is essential to design psychosocial interventions and guidelines in which the role of clinicians will be fundamental in providing information, support, and follow-up, placing the patient at the center of decision-making regarding their future, banishing paternalistic approaches.

## Figures and Tables

**Table 1:** Summary of future perspectives to face sociohealth challenges in relation to MCI diagnosis.

Future Perspective
Promote the universalization of syndromic and etiological diagnosis of MCI through the implementation of new public health policies and strategies.
2. Study of the psychosocial impact of early syndromic and etiological diagnosis of MCI in real-life clinical practice.
3. Promote comprehensive, holistic, and person-centered health and social–health care, covering the psychosocial needs detected in the reference population.

## Data Availability

Not applicable.
